# Brushing Methods for Patients Unable to Use a Mouthwash: A Preliminary Study of the Effects of Moisturizing Gel and Povidone-Iodine in Healthy Volunteers

**DOI:** 10.7759/cureus.61277

**Published:** 2024-05-28

**Authors:** Madoka Funahara, Hiromi Honda, Atsuko Nakamichi

**Affiliations:** 1 School of Oral Health Sciences, Faculty of Dentistry, Kyushu Dental University, Kitakyushu, JPN

**Keywords:** povidone iodine, moisture gel, saliva, number of bacteria, tooth brushing

## Abstract

Introduction:* *Brushing older adults or intubated patients who are unable to rinse can transmit bacteria from dental plaque into the oral cavity and increase the risk of aspiration pneumonia. Therefore, this study examined brushing methods to prevent the spread of bacteria in the oral cavity.

Methods:* *Three types of brushing methods were performed on five volunteers by dental hygienists (water group: brushing with toothbrush bristles soaked in water; gel group: brushing with a moisturizing gel placed on the toothbrush; PV-I group: brushing with toothbrush bristles dipped in povidone-iodine). Neither group spat out the saliva or gargled during brushing but brushed while wiping the water/gel/PV-I solution with a sponge brush. The same five volunteers served as subjects for the three methods. Saliva was collected before and after brushing, and the number of colonies was determined using bacterial culture.

Results:* *The water group demonstrated a significantly increased number of bacteria in the saliva owing to the spread of bacteria from the dental plaque. The gel group prevented the spread of the bacteria. The PV-I group showed a significant decrease in the number of bacteria in the saliva after brushing.

Conclusions:* *Brushing with toothbrush bristles dipped in a povidone-iodine solution is recommended for intubated or older adult patients who cannot gargle.

## Introduction

The human oral cavity contains many microorganisms (such as bacteria and fungi) that are controlled by oral cleansing methods such as oral intake, saliva secretion, and swallowing. However, bacterial counts are markedly increased in older adults with decreased oral functions, such as in cases of tongue pressure [1], fasting, or tube feeding [2], and in intubated patients [3,4]. The most effective method to reduce the oral bacterial count is rinsing the mouth, which is not possible in patients with decreased swallowing function or under intubation [5]. The increase in oral bacteria and deterioration of the patient's general condition owing to underlying diseases can cause oral bacteria to enter the lower respiratory tract along the intubation tube in intubated patients, resulting in ventilator-associated pneumonia (VAP) [6].

Oral application of 0.12% chlorhexidine (CHX) is the standard practice to prevent VAP in many countries; however, in Japan, the use of CHX in the oral cavity has been banned due to problems with anaphylactic shock in the past when CHX was used on mucous membranes. Disinfectants approved by insurance in Japan for disinfectant action are PV-I and benzethonium chloride. PV-I has a broad bactericidal spectrum, has long been used for disinfection to prevent wound infection during surgical procedures, and is expected to be a useful alternative to CHX due to its high safety profile. Although the intraoral application of povidone-iodine (PV-I) instead of CHX prevents an increase in the oral bacterial count for approximately 3 hours [7], PV-I is not commonly used in the oral care of intubated patients. Several randomized clinical trials have been conducted on the preventive effect of toothbrushes on VAP. However, many studies reported no additive effect of brushing in combination with CHX application [8-10]. Thus, although tooth brushing has no direct inhibitory effect on VAP, patients who remain intubated for long periods may develop exacerbations of periodontal disease if tooth brushing is not performed, and the risk of developing blood-borne infections in multiple organs increases owing to the presence of periodontal bacteria and cytokines. Therefore, regular tooth brushing is necessary to eliminate dental plaque, especially in patients intubated for prolonged periods or in older adults who cannot brush or rinse their mouths by themselves and require nursing care.

However, brushing may transmit plaque-derived bacteria on tooth surfaces to the oral cavity, resulting in an increased risk of pneumonia (oral care-associated pneumonia). Although rinsing with mouthwash after tooth brushing is essential, no studies have reported effective brushing methods for patients who cannot rinse their mouths. Our study aimed to determine whether using a moisturizing gel or PV-I in combination with tooth brushing can prevent the transmission of bacteria into the saliva. We also attempted to establish a safe and effective brushing method for patients who cannot rinse their mouths.

## Materials and methods

Study design and participants

This crossover study included healthy volunteers. The participants were recruited from Kyushu Dental University, Japan. The inclusion criteria were healthy adults who agreed to participate in the study, and the exclusion criteria were those who could not rinse their mouths with mouthwash. Five healthy female volunteers participated in this study. The date of their first registration was March 28, 2021. 

Method of tooth brushing

The participants underwent the following interventions without tooth brushing for 30 hours: First, the Oral Hygiene Index-Debris Index score [11] was measured. A dental hygienist performed three different brushing methods on the participants. After a one-week washout period, the remaining two brushing methods were sequentially performed as described below.

1) Water group (n=5): The toothbrush bristles were soaked in tap water to moisten them, and every quadrant was brushed for 15 s. The toothbrush bristles were then cleaned and soaked in tap water, followed by brushing.

2) Gel group (n=5): 1 g of oral care gel (OKUCHI WO ARAU GEL, Nippon Shika Yakuhin Co., Ltd., Yamaguchi, Japan) [12] was placed on the toothbrush bristles and every quadrant was brushed for 15 s. The toothbrush bristles were washed, and brushing was repeated for every quadrant after repeated washing of the toothbrush bristles and placing the gel on the toothbrush bristles. During brushing, a sponge brush was used to remove the contaminated gel containing dental plaque.

3) PV-I group (n=5): The toothbrush bristles were dipped in PV-I mouthwash solution diluted to the concentration of the mouthwash according to the manufacturer's instructions (concentration at the time of use: 0.47%), and every quadrant was brushed for 15 s. Similarly, the toothbrush bristles were rinsed after brushing each quadrant, dipped in PV-I, and brushed again.

In all groups, the patients were prohibited from spitting saliva or rinsing their mouths while brushing, and each group brushed and wiped their mouths with a sponge brush.

Collecting saliva samples and bacterial culture

Saliva samples were collected twice (before and after brushing), and the mouth was cleaned with a sponge brush. Approximately 1 mL of saliva was collected from each patient.

The saliva samples were diluted 100, 1000, and 5000 times with saline.; 100 µL of the diluted saliva samples were added to the Brain Heart Infusion medium (EIKEN CHEMICAL Co., Ltd., Tokyo, Japan) and incubated aerobically at 37°C for 48 h. Colonies on the medium were counted, and the value was multiplied by the dilution factor to give the number of colonies. 

Statistical analysis

Statistical analyses were performed using IBM Corp. Released 2019. IBM SPSS Statistics for Windows, Version 26.0. Armonk, NY: IBM Corp. The number of bacteria in the saliva before and after brushing in each group was compared, and the differences were tested using the paired-samples t-test. A p-value <0.05 was considered statistically significant.

## Results

Table 1 shows the background characteristics of the five volunteers who participated in this study.

**Table 1:** Participant characteristics

Variable		Number of participants/means ± SD
Sex	male	0
	female	5
Age	years	25±8.94
Number of teeth		28±1.41
Oral Hygiene Index-Debris Index score	Water	1.51±0.22
	Gel	1.37±0.25
	PV-I	1.50±0.50
Bacterial count Before brushing (logCFU/mL)	Water	6.71±0.41
	Gel	6.88±0.57
	PV-I	6.77±0.32

Water group

**Figure 1:**
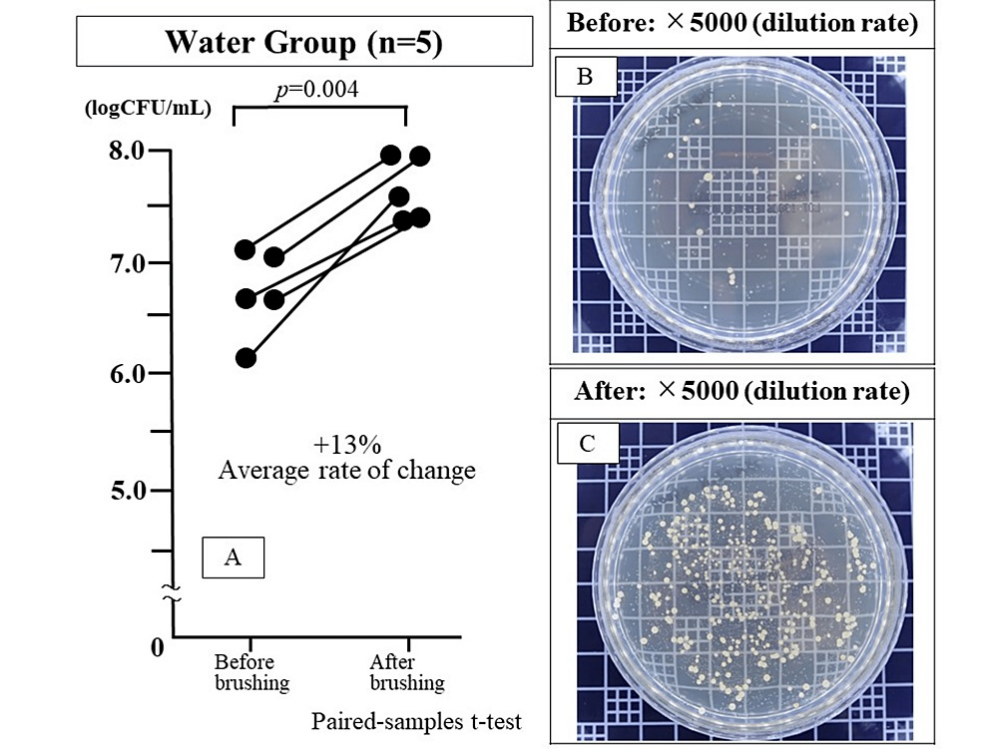
Bacterial counts in the saliva before and after brushing in the water group The number of bacteria significantly increased after tooth brushing (*p*=0.004: Paired-samples t-test). Label A is a graph of bacteria counts before and after brushing. Label B is a photograph of a salivary bacteria culture petri dish before brushing (5000x dilution). Label C is a photograph of the bacteria culture petri dish after brushing (5000x dilution).

The logarithm of the number of bacteria in the saliva before and after brushing was 6.71 ± 0.42 and 7.62 ± 0.32, respectively. The number of bacteria in the saliva markedly increased in all five cases. The mean increase rate was +13.6%, with a significant difference in bacterial counts before and after brushing (p=0.004) (Figure 1).

Gel group

**Figure 2:**
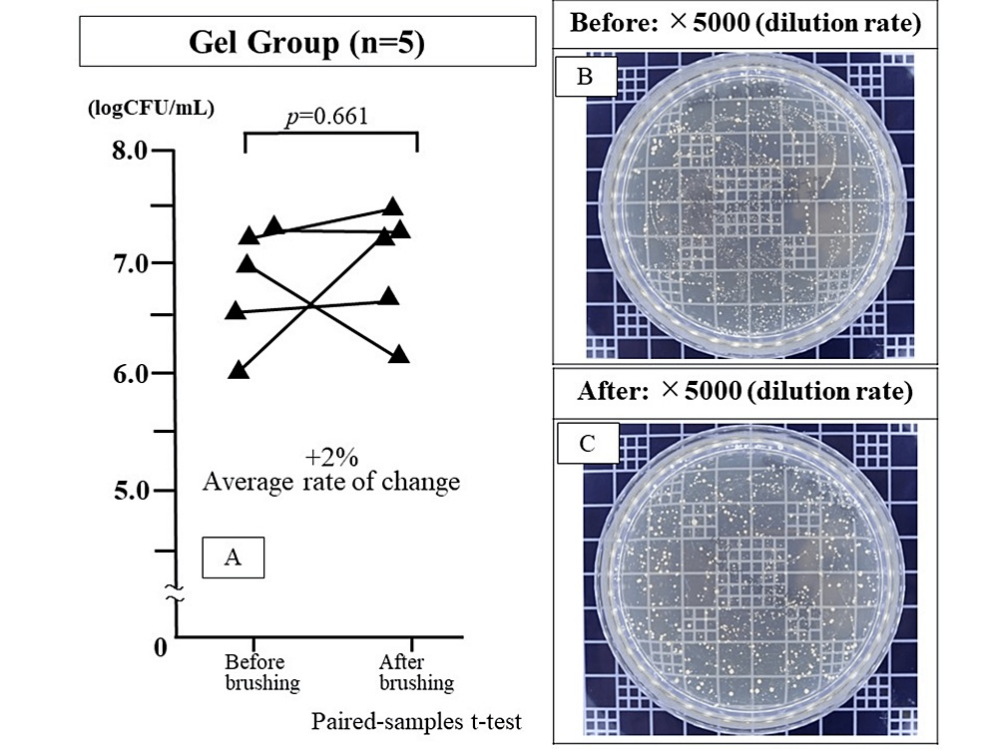
Bacterial counts in the saliva before and after brushing in the gel group Salivary bacterial count after brushing showed minimal change compared to that before brushing (p=0.661: Paired-samples t-test). Label A is a graph of bacteria counts before and after brushing. Label B is a photograph of a salivary bacteria culture petri dish before brushing (5000x dilution). Label C is a photograph of the bacteria culture petri dish after brushing (5000x dilution).

The logarithm of the number of bacteria in saliva before and after brushing was 6.88 ± 0.57 and 7.03 ± 0.52, respectively. The number of bacteria in the saliva increased in three participants and decreased in two participants. The mean increase rate was +2.2%, and no significant difference was observed in the bacterial counts before and after brushing (p=0.661) (Figure 2).

PV-I group

**Figure 3:**
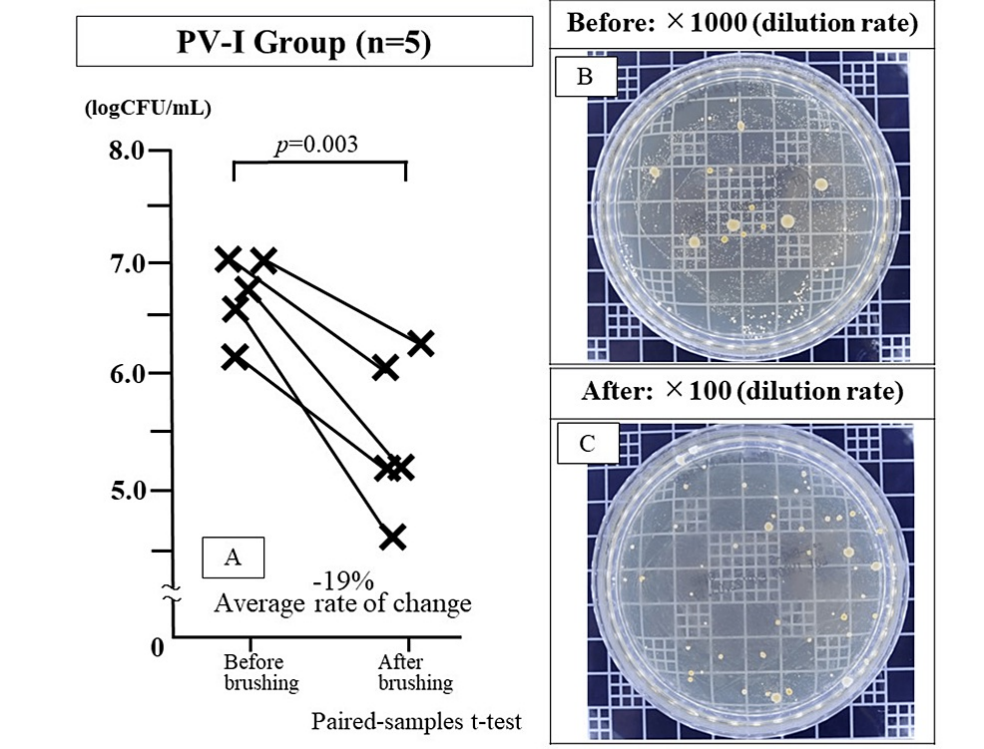
Bacterial counts in the saliva before and after brushing in the povidone-iodine (PV-I) group Salivary bacterial count after brushing was significantly lower than that before brushing (*p*=0.003: Paired-samples t-test). Label A is a graph of bacteria counts before and after brushing. Label B is a photograph of a salivary bacteria culture petri dish before brushing (1000x dilution). Label C is a photograph of the bacteria culture petri dish after brushing (100x dilution).

The logarithm of the number of bacteria in the saliva before and after brushing was 6.77 ± 0.32 and 5.50 ± 0.61, respectively. All patients exhibited a marked decrease after brushing. The mean increase rate was -18.8%, with a significant bacterial count reduction before and after brushing (*p*=0.003) (Figure 3).

**Figure 4:**
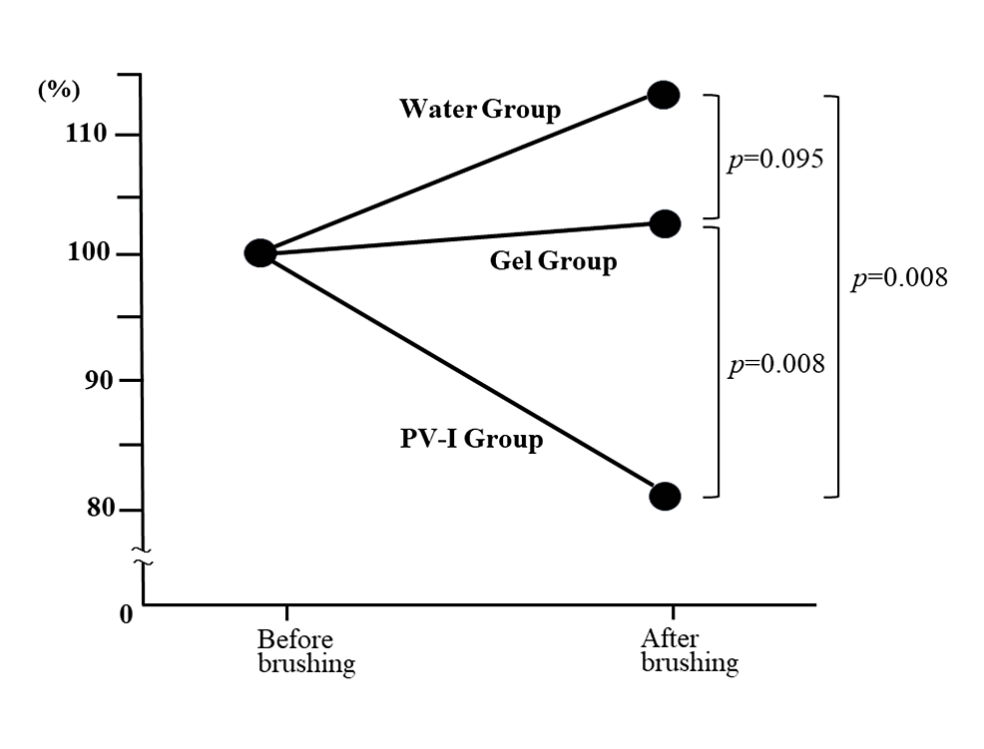
Comparison of bacterial reduction rates between the three groups Mann-Whitney U test, Bonferroni method (significant difference at p<0.0167)

Changes in bacterial counts after brushing in each group were compared. Taking the number of bacteria before brushing as 100%, the number of bacteria after brushing was 113.7% in the water group, 102.6% in the gel group, and 81.1% in the PV-I group, showing a significant decrease in the PV-I group compared to the other two groups (Figure 4).

## Discussion

Aspiration pneumonia occurs when the aspiration of saliva-containing pathogenic microorganisms is combined with a weakened immune system and deteriorating general health. The risk of aspiration pneumonia is particularly high in older adults with impaired swallowing function and in patients who have undergone highly invasive surgeries such as cancer. It is important to reduce the number of bacteria in saliva, prevent aspiration, and improve the general condition of the patient to prevent aspiration pneumonia. Therefore, the importance of oral care for older adults and postoperative patients is widely reported [1-4,13-17]. Additionally, VAP in endotracheally intubated patients is directly related to survival outcomes and is a serious post-operative complication [6].

Oral application of 0.12% CHX is generally used to prevent VAP; however, its use in the oral cavity is prohibited in Japan owing to anaphylactic shock upon using CHX on the mucous membrane. Therefore, mechanical cleaning techniques such as tooth brushing are widely employed in intubated patients in clinical practice. Brushing is ineffective in preventing VAP [8-10] and can contribute to an increase in the number of bacteria in the oropharyngeal fluid by promoting the spread of bacteria from the plaque into the oral cavity, which induces VAP. However, it is imperative to remove dental plaque (especially in long-term intubated patients) by brushing to prevent new sources of infection owing to plaque adhesions, such as periodontal disease. Therefore, a brushing method that does not increase the number of bacteria in the oropharyngeal fluid should be established.

In this study, the number of bacteria in the saliva markedly increased after tooth brushing, and the increase in the bacterial count slightly decreased when wiping was used in combination with brushing. However, the spread of bacteria could not be completely prevented and was only eliminated when brushing was followed by rinsing with mouthwash [5]. However, no studies have reported effective brushing methods for patients who are unable to rinse their mouths. Moriya et al. developed a new oral moisturizing gel and reported that the spread of bacteria from dental plaque during brushing could be prevented using this gel for tooth brushing [18]. Therefore, we compared the number of bacteria in the saliva after brushing with a gel to that after normal brushing. The number of bacteria in the saliva increased by 13.6% after normal brushing, whereas the rate of increase in bacterial counts was reduced to 2.2% when the gel was used for tooth brushing. This could be attributed to the fact that the plaque that was removed from the teeth during brushing was trapped by the gel, thereby preventing it from reaching the oral cavity. In contrast, the number of bacteria in the saliva after brushing was reduced by 18.8% when the toothbrush bristles were soaked in PV-I rinsing solution diluted to the recommended concentration, compared with that before brushing. This could be attributed to the bactericidal effect of PV-Ion on dental plaque; thus, most of the bacteria that reached the oral cavity during brushing were non-viable. These findings suggest that soaking toothbrush bristles in a PV-I solution before brushing may help prevent pneumonia in intubated patients.

Our study had several limitations. First, the sample size was small; hence, the generalizability of the results is questionable. Second, the participants were healthy individuals with relatively good oral hygiene who stopped brushing their teeth for 30 hours; thus, the amount of plaque adhering to their teeth was low. The amount of PV-I soaked in the toothbrush was sufficient to disinfect bacteria in the diffused plaques of healthy volunteers. However, it is unknown whether a similar concentration is sufficient to disinfect bacteria in the plaques of patients with poor oral hygiene and high plaque volume. Nevertheless, this is the first study demonstrating that brushing with PV-I is useful for eliminating plaques and reducing the spread of viable bacteria into the oral cavity of patients who cannot rinse their mouth. Further studies should be conducted on intubated patients or older adults who cannot rinse their mouths and require nursing care to validate the findings of this study.

## Conclusions

Brushing older adults or intubated patients who are unable to rinse can transmit bacteria from dental plaque into the oral cavity and increase the risk of aspiration pneumonia. In this study, we examined changes in salivary bacterial counts after three different brushing methods. Brushing with water and a toothbrush, combined with wiping, significantly increased the number of bacteria in the saliva, owing to the spread of bacteria in the dental plaque. Brushing with a toothbrush and bristles dipped in a moisturizing gel prevented the spread of bacteria. Brushing with a toothbrush with bristles dipped in PV-I significantly decreased the number of bacteria in the saliva after brushing. Therefore, we believe that brushing with toothbrush bristles dipped in a PV-I solution is a safe and effective method for patients who cannot rinse their mouths.

## References

[1] Funahara M, Soutome S, Sakamoto Y (2023). Relationship between tongue pressure and salivary bacteria in the older adults requiring long-term care. Gerontology.

[2] Sakamoto Y, Tanabe A, Moriyama M (2022). Number of bacteria in saliva in the perioperative period and factors associated with increased numbers. Int J Environ Res Public Health.

[3] Funahara M, Hayashida S, Sakamoto Y, Yanamoto S, Kosai K, Yanagihara K, Umeda M (2015). Efficacy of topical antibiotic administration on the inhibition of perioperative oral bacterial growth in oral cancer patients: a preliminary study. Int J Oral Maxillofac Surg.

[4] Hayashida S, Funahara M, Sekino M (2016). The effect of tooth brushing, irrigation, and topical tetracycline administration on the reduction of oral bacteria in mechanically ventilated patients: a preliminary study. BMC Oral Health.

[5] Funahara M, Yamaguchi R, Honda H, Matsuo M, Fujii W, Nakamichi A (2023). Factors affecting the number of bacteria in saliva and oral care methods for the recovery of bacteria in contaminated saliva after brushing: a randomized controlled trial. BMC Oral Health.

[6] Chastre J, Fagon JY (2002). Ventilator-associated pneumonia. Am J Respir Crit Care Med.

[7] Tsuda S, Soutome S, Hayashida S, Funahara M, Yanamoto S, Umeda M (2020). Topical povidone iodine inhibits bacterial growth in the oral cavity of patients on mechanical ventilation: a randomized controlled study. BMC Oral Health.

[8] Munro CL, Grap MJ, Jones DJ, McClish DK, Sessler CN (2009). Chlorhexidine, toothbrushing, and preventing ventilator-associated pneumonia in critically ill adults. Am J Crit Care.

[9] Pobo A, Lisboa T, Rodriguez A (2009). A randomized trial of dental brushing for preventing ventilator-associated pneumonia. Chest.

[10] Lorente L, Lecuona M, Jiménez A (2012). Ventilator-associated pneumonia with or without toothbrushing: a randomized controlled trial. Eur J Clin Microbiol Infect Dis.

[11] Greene JC, Vermillion JR (1960). The oral hygiene index: a method for classifying oral hygiene status. J Am Dent Assoc.

[12] (2024). Nippon Shika Yakuhin: OKUCHI WO ARAU GEL: Summary of product characteristics (in Japanese). https://www.nishika.co.jp/upfiles/53_pdf_8/OG-A-5.pdf.

[13] Yoneyama T, Yoshida M, Ohrui T (2002). Oral care reduces pneumonia in older patients in nursing homes. J Am Geriatr Soc.

[14] Soutome S, Yanamoto S, Funahara M (2017). Effect of perioperative oral care on prevention of postoperative pneumonia associated with esophageal cancer surgery: A multicenter case-control study with propensity score matching analysis. Medicine (Baltimore).

[15] Soutome S, Hasegawa T, Yamguchi T (2020). Prevention of postoperative pneumonia by perioperative oral care in patients with esophageal cancer undergoing surgery: a multicenter retrospective study of 775 patients. Support Care Cancer.

[16] Iwata E, Hasegawa T, Yamada SI (2019). Effects of perioperative oral care on prevention of postoperative pneumonia after lung resection: Multicenter retrospective study with propensity score matching analysis. Surgery.

[17] Ishimaru M, Matsui H, Ono S, Hagiwara Y, Morita K, Yasunaga H (2018). Preoperative oral care and effect on postoperative complications after major cancer surgery. Br J Surg.

[18] Moriya M, Matsuyama M, Inukai J (2016). A new oral care gel to prevent aspiration during oral care. Nihon Ronen Igakkai Zasshi.

